# Generation of Human B-Cell Lines Dependent on CD40-Ligation and Interleukin-4

**DOI:** 10.3389/fimmu.2015.00055

**Published:** 2015-02-11

**Authors:** Jacques Banchereau

**Affiliations:** ^1^Jackson Laboratory for Genomic Medicine, Farmington, CT, USA; ^2^Vaccine Research Institute, U955, Hopital Henri Mondor, Creteil, France

**Keywords:** human B lymphocytes, isotype switch, plasma cells, memory B-cells, IL-4

## Where We Started

The description of factor-dependent cytotoxic T-cell lines in the late 1970s transformed T-cell biology (1). Among other events, it led to the cloning of a cDNA encoding IL-2 (2). It also led to the identification of T-cell subsets and formulation of the Th1/Th2 concept in the 80s (3). However, comparable advances in B-cell biology were lacking, partly because of the lack of availability of factor-dependent B-cell lines. This was the case despite the fact that B-cell-specific trophic factors, including BSF (B-cell stimulation Factor), BCGF (B-cell growth factor), and BCDF (B-cell differentiation factor) had been described in the supernatants of activated T cells.

The cloning at DNAX, our sister institute acquired by Schering–Plough, of a cDNA encoding BSF-1, later renamed IL-4, in mouse (4) and in human (5) was a first step forward to the definition of the molecules controlling B-cell growth and differentiation. In our laboratory, based in Dardilly near Lyon (France), we found that cultured purified human B-cells triggered with anti-B-cell receptor (BCR) and IL-4 resulted in significant B-cell proliferation as measured by tritiated thymidine counts, a common way of measuring B-cell proliferation in the 1980–1990s (6). These cultures yielded more B-cells than did naïve cultures or those exposed to anti-BCR alone or IL-4 alone. Yet, these cultures established with anti-BCR plus IL-4 yielded less viable B-cells than were input. Thus, we, B-cell biologists had not yet been able to reproduce with B-cells the factor-dependent growth of T cells that our colleagues T-cell biologists have been able to achieve.

## Feeder Cells and New Monoclonal Antibodies Yield More Robust B-Cell Cultures

A possible explanation for our lack of success was the absence of feeder cells, which had become part of the T-cell culture system and proved necessary to allow for the expansion of human T-cell lines and clones. Meanwhile, Kevin Moore and his colleagues at DNAX, cloned a human cDNA coding for FcγRII/CD32 and found that FcγRII/CD32-transfected fibroblast cell lines could present monoclonal antibodies in a manner that allowed for cross-linking of the target molecule of the relevant cell (7, 8). More specifically, antibodies to the T-cell CD3 complex presented by these transfected cells together with IL-2 could induce prolonged T-cell proliferation (9). Thus, we wondered whether the presentation of monoclonal antibodies specifically directed at B-cell surface molecules in the presence of B-cell tropic cytokines would lead to the proliferation and expansion of B-cells.

By the end of the 80s, we, investigators from Schering–Plough/DNAX had cloned cDNAs encoding human GM-CSF (10), IL-4, IL-5 (5, 6), and FcgR/CD32 (8). We had also generated a number of monoclonal antibodies that would recognize B-cells including a CD40 antibody (11) and an anti-B7 antibody now known as CD86 (12). When Paolo de Paoli came to our lab to perfect his flow cytometry skills, he took a side project to refine methods for culturing sorted B-cells using both classical and new approaches, including the addition of a feeder-layer of CD32/FcγR-transfected cells as discussed above (9).

To this end, 96-well-plate microwells were first seeded with the irradiated fibroblast line. A few thousand B-cells were then added along with a few selected monoclonal antibodies with or without IL-2 or IL-4. Cultures were harvested 3–5 days later after a brief pulse with tritiated thymidine. It very quickly became apparent that the combination of the CD40 antibody Mab 89 (11) and IL-4 could induce unusually strong B-cell proliferation. The well-known CD40 antibody G28-5 made by Ed Clark and Jeff Ledbetter also proved highly effective in this system (13, 14). Curiously, IL-2 was unable to enhance CD40-induced B-cell proliferation, although it did enhance the proliferation of B-cells activated through their BCR. Furthermore, the fibroblast layer provided some feeder effect, as cross-linking the CD40 antibody on plastic was never as effective in inducing prolonged B-cell proliferation as presenting it with the CD32-transfected fibroblast.

## New System Increased B-Cell Proliferation and Enabled Long-Term B-Cell Culture and Studies of B-Cell Differentiation

The next critical experiment was to determine whether these culture conditions actually increased the output of B-cells. Indeed, it was very rewarding to find that the cultures made with CD40 antibody and IL-4 did generate more B-cells than were initially seeded. Subsequent experiments showed that with this new method we could establish proliferative B-cell cultures using relatively low numbers of B-cells (5,000 or less per well) compared to our previous purified B-cell cultures triggered with anti-BCR (20,000–50,000 per well). This important finding, however, was not the end of the story, as we still had to show that this novel B-cell culture system would allow for the long-term growth (i.e., at least 3 weeks), of B-cells following splitting and feeding.

Some human B-cells harbor the Epstein–Barr virus (EBV), which, upon reactivation, can induce the generation of factor-independent lymphoblastoid B-cell lines. Thankfully, removal of the CD40 antibody and IL-4 quickly resulted in B-cell death. Furthermore, the factor-dependent B-cell lines failed to express EBNA-2 (Epstein–Barr Nuclear Antigen 2). These two findings led us to conclude that we were indeed generating factor-dependent human B-cell lines (15).

As is so often the case, novel methodologies enable us to address a whole new set of questions. We were thus wondering whether this new, feeder-layer/monoclonal antibody-based culture system would permit us to mimic many of the events happening in the germinal center where isotype switch, somatic mutations and differentiation into either memory B-cells or plasma cells are thought to occur (16). Indeed, Yong-Jun Liu and Ian McLennan showed that CD40-ligation prevents the spontaneous apoptosis of human centrocytes, which undergo antigen-driven selection within germinal centers (17). Our later studies and those from others demonstrated that CD40-activated B-cells could undergo isotype switch toward IgE when exposed to IL-4 or IL-13 (18, 19). Upon exposure to IL-10, CD40-activated B-cells switch toward IgG1 and IgG3 as well as IgA1 and IgA2 (20). The combination of IL-10 and TGFβ further enhances the IgA2 response. The critical studies led with the CD40 antibodies led to the cloning of it ligand (CD40-L), a molecule transiently expressed on T cells, by investigators at Immunex (21). The importance of CD40–CD40-L interactions in isotype switching in humans was further established when patients devoid of functional CD40-L were shown not to display switched isotypes (22–25). In further studies using CD40-L-transfected fibroblasts, rather than the combination of CD32/FcγR-transfected fibroblasts and CD40 antibody, we could show that CD40-ligation induces germinal center B-cells to differentiate into memory B-cells rather than plasma cells (26). Thus, this new culture method could robustly recapitulate key features of the germinal center and thus enable greater insight into the events leading to B-cell differentiation than previous approaches.

## Exploiting the New B-Culture System for Generating Human Monoclonal Antibodies

A practical application of the CD40-system has been the efficient generation of human monoclonal antibodies. We eventually simplified the system to the point where peripheral blood cells (about 5,000 per well) from individuals displaying selected antibody specificities in their serum were simply cultured over CD40-L-expressing fibroblasts in the presence of exogenous EBV. Analysis of culture supernatants after 10 days eventually revealed the presence of antibodies of the desired specificity. This allowed us to generate large a number of monoclonal antibodies, such as those against the Bullous Pemphigoid Antigen1 (27), and against allergens, such as those from birch pollen (28). Nearly all these lines proved to be easy to generate; however, one set of autoantibodies gave us a hard time: autoantibodies against IL1α, which we previously found were expressed in about 10% of the healthy population. We generated dozens of B-cell lines producing anti-IL1α antibodies but had difficulty cloning them. We eventually isolated a single clone (29) that produced a high affinity (Kd~10^−10^) neutralizing monoclonal antibody. The reason why the cloning of the lines generating these monoclonal antibodies suggested to us that IL1α might have a critical role in B-cell expansion, an hypothesis we could not confirm. A strategic decision by the Schering–Plough leadership resulted in the discontinuation of our human monoclonal antibody program in 1994 around the time that the FDA rejected approval of the anti-sepsis monoclonal antibody, Nebacumab, also known as Centoxin^®^ (30). Today, however, human monoclonal antibodies are a major success in the biotechnology and pharmaceutical world. Several methodologies to generate monoclonal antibodies are currently in use. These include: (1) engineering of mouse monoclonal antibodies, (2) generating monoclonal antibodies from animals whose Ig locus has been swapped with a human Ig locus, (3) the use of phage display libraries from pools of memory B-cells, (4) a method similar to ours where CD40-L cells were replaced by CpG (31), and (5) the isolation of single B-cells and the cloning of their heavy and light chains (32). Other have taken advantage of the power of the “CD40-system” to generate long-term B-cell lines that could be used as highly efficient antigen-presenting cells to generate autologous antigen-specific T cells for adoptive immunotherapy (33). The system can even permit the establishment of long-term porcine B-cell cultures (34). It is important to know that CD40 is expressed by a number of cell types other than B-cells. Most importantly dendritic cells express CD40 and are activated upon CD40-ligation (35). A few other cell types do as well [see Ref. (36)].

In conclusion, this study showed that it was possible to grow B-cells like T cells and opened up a path toward obtaining human monoclonal antibodies and understanding B-cell signaling. Before this work, many labs had tried to get human monoclonal antibodies from EBV transferred cells but the yields were small and most often low affinity IgM were generated. I am indebted in the numerous students and colleagues from Schering–Plough and DNAX who worked with me on these various projects. The support of Schering–Plough was essential for these studies.

## Conflict of Interest Statement

The author declares that the research was conducted in the absence of any commercial or financial relationships that could be construed as a potential conflict of interest.
